# Post‐Stroke Depression Is Associated With Shared Neurodevelopmental Risk and Circuit Disruption

**DOI:** 10.1002/brb3.71502

**Published:** 2026-05-24

**Authors:** Zhi‐Jie Xu, Su‐Xiang Zhang, Ji‐Ling Li, Jia‐Jia Wu, Jie Ma, Zhen‐Zhen Ma, Ji‐Ming Tao, Xu‐Yun Hua, Jian‐Guang Xu

**Affiliations:** ^1^ School of Rehabilitation Science Shanghai University of Traditional Chinese Medicine Shanghai China; ^2^ Engineering Research Center of Traditional Chinese Medicine Intelligent Rehabilitation Ministry of Education Shanghai China; ^3^ Department of Rehabilitation Shuguang Hospital Affiliated to Shanghai University of Traditional Chinese Medicine Shanghai China; ^4^ Department of Traumatology and Orthopedics Yueyang Hospital of Integrated Traditional Chinese and Western Medicine Shanghai China; ^5^ Center of Rehabilitation Medicine Yueyang Hospital of Integrated Traditional Chinese and Western Medicine Shanghai University of Traditional Chinese Medicine Shanghai China

**Keywords:** default mode network, neurodevelopment, post‐stroke depression, shared genetic risk

## Abstract

**Introduction:**

Post‐stroke depression (PSD) affects approximately 30% of stroke survivors and worsens functional outcomes, yet its biological basis remains poorly understood.

**Methods:**

We integrated cross‐disorder genomics, developmental spatial transcriptomics, and causal neuroimaging in 16 PSD patients and 14 matched controls. Polygenic overlap was quantified using MiXeR and conditional FDR (condFDR). Shared risk variants were mapped using genetically informed spatial mapping of cells for complex traits (gsMap) onto the embryonic mouse brain (E16.5). Resting‐state fMRI with group independent component analysis (ICA), functional network connectivity (FNC), and spectral dynamic causal modeling (spDCM) characterised circuit‐level alterations.

**Results:**

Stroke and depression showed robust polygenic overlap (Dice = 0.09). condFDR prioritized five pleiotropic loci implicating HDAC9‐mediated neurovascular inflammation and PITX2‐driven cardio‐cerebral signaling. gsMap revealed enrichment of shared genetic risk in developing cortical regions at E16.5. PSD exhibited selective default mode network (DMN)–sensorimotor network (SMN) decoupling and reduced directed DMN→SMN influence (BPA = –0.12 Hz; *Pp* > 0.99). Auditory network (AN) outflow to DMN, SMN, and ventral attention network (VAN) was broadly attenuated, and AN→DMN effective connectivity scaled with depressive severity (BPA = –0.19 Hz; *Pp* > 0.99).

**Conclusion:**

PSD reflects a unified developmental–acquired pathophysiology where latent developmental genetic vulnerability, revealed at E16.5, is unmasked by stroke‐triggered circuit decompensation. HDAC9/PITX2 pathways and AN–DMN circuitry are mechanistically grounded targets for potential biomarker development and circuit‐informed interventions.

## Graphical abstract

Genetic vulnerability and stroke‐related circuit disruption may jointly contribute to post‐stroke depression. Altered auditory–default mode network connectivity was associated with symptom severity, suggesting a potential circuitlevel biomarker.

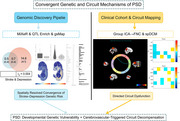

## 1 Introduction

Stroke is a leading cause of death and disability worldwide (Q. Zhang et al. 2026), with consequences extending beyond motor deficits to include neuropsychiatric sequelae (GBD 2021 Stroke Risk Factor Collaborators 2024). Pos‐tstroke depression (PSD), affecting approximately 30% of survivors, is the most prevalent complication and independently predicts impaired recovery (Mensah et al. 2023), reduced quality of life, and elevated all‐cause mortality (Lanctôt et al. 2020). Although both stroke and depression are heritable, the architecture and developmental anchoring of their shared genetic risk remain poorly defined (Gomberg et al. 2024). Crucially, it is unknown how stroke disrupts directed brain network interactions to precipitate depression, and whether such circuit‐level dysfunction colocalizes with genetic risk patterns established during early brain development (Wu et al. 2024). Resolving this gap is essential for a mechanism‐based understanding of PSD pathogenesis.

PSD arises not from focal lesions but from widespread reorganization of large‐scale brain networks due to ischemic injury. Neuroimaging shows that even localized strokes alter intrinsic connectivity, particularly within the default mode network (DMN), sensorimotor network (SMN), and auditory network (AN) (Q. Lu et al. 2025). This cross‐network imbalance represents a key neural mechanism of PSD (Bisogno et al. 2025). However, most studies rely on undirected functional connectivity, leaving network‐level causal architecture uncharacterized. It remains unknown how stroke perturbs directional influence among network nodes (i.e., effective connectivity) and whether such aberrant interactions underlie depressive symptom emergence and severity (Wiemer et al. 2025). Resolving this question is essential for defining circuit‐level PSD pathophysiology.

The genetic architecture linking stroke and depression remains unmapped onto developmentally relevant neural substrates. Despite advances in large‐scale genomics and biobanks, it remains unclear how shared risk variants regulate gene expression via expression and splicing quantitative trait loci (eQTLs and sQTLs) in disease‐relevant brain regions (C. Li et al. 2025). Conventional approaches cannot bridge these scales. We address this gap using an integrative framework unifying cross‐disorder genetics, spatially anchored genetic effects, and directed brain network dynamics (van den Heuvel et al. 2019).

To address this challenge, we used MiXeR and conditional false discovery rate (condFDR) to identify pleiotropic loci shared between stroke and depression (Frei et al. 2019; Smeland et al. 2020). We mapped variants onto tissue‐specific regulatory architectures using eQTL/sQTL data and genetically informed spatial mapping of cells for complex traits (gsMap)‐based spatial enrichment in the E16.5 mouse embryonic brain, and implemented a hierarchical neuroimaging pipeline to characterize undirected functional network connectivity (FNC) and probe causal interactions via spDCM (Friston et al. 2014). This cascade—from shared polygenic risk to developmentally patterned vulnerability to acquired circuit failure—defines a mechanistic continuum underlying PSD.

## 2 Materials and Methods

### 2.1 GWAS Inclusion and Quality Control

Genome‐wide association study (GWAS) summary statistics for stroke were drawn from a large‐scale meta‐analysis of 521,612 individuals of European ancestry (Malik et al. 2018), and those for depression from a UK Biobank–based study of 394,626 European‐ancestry participants (Loya et al. 2025). Prior to integrative analysis, we applied a unified quality control (QC) pipeline: (i) exclusion of variants with minor allele frequency (MAF) < 0.01 to enhance statistical power and mitigate multiple testing burden; and (ii) removal of all single‐nucleotide polymorphisms (SNPs) within the major histocompatibility complex (MHC) region (chr6: 25–35 Mb) to eliminate spurious associations driven by its extreme linkage disequilibrium (LD) structure.

### 2.2 Participants

**FIGURE 1:**
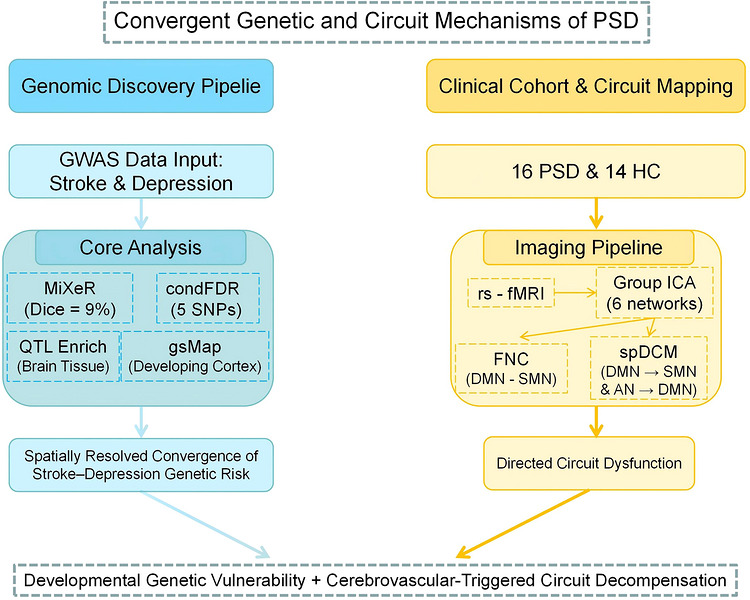
The study flow chart. AN, auditory network; condFDR, conditional false discovery rate; DMN, default mode network; FNC, functional network connectivity; Group ICA, group independent component analysis; gsMap, genetically informed spatial mapping of cells for complex traits; GWAS, genome ‐ wide association study; HC, healthy controls; MiXeR, mixture of eXperts for cross‐disorder analysis; PSD, post ‐ stroke depression; QTL Enrich, quantitative trait locus enrichment; rs‐fMRI, resting‐state functional MRI; SMN, sensorimotor network; spDCM, spectral dynamic causal modeling.

We enrolled 16 patients with PSD and 14 age‐, sex‐, and years of education‐matched healthy controls (HC). PSD cases were recruited from Shuguang Hospital Affiliated to Shanghai University of Traditional Chinese Medicine, and were independently diagnosed by two board‐certified rehabilitation physicians per the Chinese Clinical Practice Guideline for PSD (F.‐Y. Zhao et al. 2018). HC participants were community‐recruited via public notices and online platforms. All participants were aged 18–80 years, cognitively intact, and free of MRI contraindications, centrally acting medications, and a history of chronic alcohol/substance abuse. PSD‐specific criteria included: age 40–80 years; absence of impaired consciousness, aphasia, or severe organ dysfunction; mild‐to‐moderate depression (Hamilton Depression Rating Scale, HDRS 8–24); and written informed consent. Exclusion criteria comprised active suicidal risk, stroke onset < 2 weeks prior, recurrent or treatment‐resistant depression, comorbid psychiatric disorders, or investigator‐determined unsuitability. Demographic data (age, sex, and education) were collected pre‐scan. The study was approved by the Shuguang Hospital Ethics Committee (No. 20241419‐002‐01), conducted in accordance with the Declaration of Helsinki, and registered prospectively in the Chinese Clinical Trial Registry (ChiCTR2400085211; June 3, 2024). Written informed consent was obtained from all participants (Figure 1).

### 2.3 Cross‐Disorder Genetic Architecture and Conditional Loci Discovery

We quantified the shared genetic architecture between stroke and depression using MiXeR (Frei et al. 2019), a Bayesian mixture‐modeling framework that integrates GWAS summary statistics with a European‐ancestry LD reference panel (approximately 11 million SNPs). MiXeR estimates trait‐specific and shared burdens of variants, polygenicity, SNP‐based heritability, and effect concordance, and summarizes overall overlap via a standardized Dice coefficient. To boost the discovery of comorbidity‐relevant loci, we applied condFDR analysis. This empirical Bayes framework leverages auxiliary GWAS summary statistics to reweight *p*‐values, enhancing power to detect SNPs whose association with the primary trait is conditionally enriched by the secondary trait. Shared loci were operationally defined using a threshold of condFDR < 0.01, which aligns with recent cross‐trait genetic studies demonstrating that this cutoff optimally balances false‐discovery control and sensitivity for identifying pleiotropic signals in highly polygenic architectures (Smeland et al. 2020; Y. Zhang et al. 2026; Q. Zhao et al. 2025). For functional annotation and gene prioritization via FUMA, we included all SNPs with *p* < 5 × 10^−7^ in the primary GWAS, ensuring comprehensive coverage of sub‐threshold signals enriched by condFDR.

### 2.4 Tissue‐Specific Enrichment of Genetic Risk

To characterize tissue‐level regulatory enrichment of stroke and depression risk, we performed enrichment analyses using QTLEnrich (M. Liu et al. 2024), which assesses over‐representation of GWAS signals among eQTLs and sQTLs across 49 GTEx v8 tissues. The analysis accounted for key genomic confounders, including MAF, distance to transcription start sites, and local LD. Enrichment was quantified as adjusted fold enrichment, with significance evaluated using *p*‐values. Quantile–quantile (QQ) plots were additionally examined to assess global signal integrity.

### 2.5 Spatial Mapping of Genetic Risk Across Cell Types and Anatomical Niches

To resolve the neuroanatomical and cellular context of shared genetic risk, we applied gsMap—a genetically informed, cross‐species framework that projects human GWAS signals onto a spatially resolved transcriptomic atlas of the E16.5 mouse embryo (25 organs) (Song et al. 2025). By leveraging co‐expression patterns within anatomically defined cell populations, gsMap infers the cellular localization of polygenic risk even without direct human spatial data, generating dual‐resolution enrichment maps that integrate cell‐type identity and spatial position across brain and peripheral tissues. This approach anchored both shared and disorder‐specific genetic signals to precise anatomical niches, revealing spatially localized pathogenic foci with striking regional specificity and delineating convergent and divergent cellular substrates underlying stroke–depression comorbidity (Xia et al. 2025).

### 2.6 Resting‐State fMRI Acquisition and Preprocessing

Resting‐state fMRI data were acquired at a tertiary medical center using a 3.0 T Siemens Skyra scanner (20‐channel head–neck coil). A gradient‐echo EPI sequence was used (TR = 2500 ms, TE = 21 ms, flip angle = 90°, FOV = 220 × 220 mm^2^, 64 × 64 matrix, 43 contiguous axial slices, 3.2 mm thickness, no gap; anterior–posterior phase encoding), yielding 240 volumes in 10 min. Participants remained awake with eyes closed, avoiding structured thought.

Preprocessing was performed in MATLAB R2022b using SPM12 and RESTplus v1.30 (Jia et al. 2019): (i) removal of the first 10 volumes; (ii) slice‐timing correction (middle slice reference); (iii) motion correction (subjects with > 3 mm translation or > 3°rotation were excluded); (iv) spatial normalization to MNI space via the SPM EPI template, resampled to 3 × 3 × 3 mm^3^; (v) nuisance regression of Friston‐24 motion parameters, white matter, and CSF signals derived from MNI‐space tissue probability maps; (vi) band‐pass filtering (0.01–0.08 Hz); and (vii) 6‐mm FWHM Gaussian smoothing. To control for lesion laterality, all stroke patients with right‐hemisphere lesions underwent midsagittal flipping of functional and structural images, aligning all lesions to the left hemisphere prior to group‐level analysis.

### 2.7 Resting‐State Network Identification and Functional Connectivity

We applied group independent component analysis (ICA) to the full cohort using the GIFT toolbox (v4.0b) to identify population‐level resting‐state networks (RSNs) (Allen et al. 2011). The model order was automatically set to 64 independent components (ICs) via the Minimum Description Length (MDL) criterion (Majeed and Avison 2014). Decomposition used the Infomax algorithm with 500 ICASSO iterations to ensure solution stability. Subject‐specific spatial maps and time courses were derived through back‐reconstruction and converted to *z*‐scores. To objectively assign ICs to canonical systems, we performed spatial regression against eight a priori templates from the Yeo cortical parcellation—comprising the DMN, SMN, dorsal and ventral attention networks (DAN/VAN), AN, left and right frontoparietal network (lFPN/rFPN), and visual networks (VN) (Yeo et al. 2011). An IC was assigned to a network if it showed the highest standardized regression coefficient (*β*) exceeding 0.3—a threshold validated for specificity in templatematching—and exhibited peak activation within anatomically plausible regions.

FNC was then quantified using GIFT's MANCOVA framework (Calhoun et al. 2001). For each participant, pairwise Pearson correlations between all IC time courses generated a symmetric FNC matrix. Correlation coefficients were Fisher's *r*‐to‐*z* transformed to approximate normality. Group differences (PSD vs. HC) were tested per connection using two‐sample *t*‐tests, adjusting for age, sex, and years of education, with multiple comparisons corrected via FDR (*q* < 0.05) (Erhardt et al. 2011).

### 2.8 Effective Connectivity and Statistical Inference

To test whether PSD disrupts directed interactions within functional circuits, we applied spDCM as implemented in SPM12 (r7771, DCM12). Network nodes were defined as 6‐mm‐radius spherical regions of interest (ROIs) centered on peak MNI coordinates from the six canonical RSNs retained following group ICA and template matching. The spDCM model was constructed exclusively using these six networks to ensure optimal spatial specificity and analytical consistency. For each participant, the first eigenvariate of the preprocessed BOLD time series—regressed for white matter and cerebrospinal fluid signals—was extracted from each ROI (Kita et al. 2025). A full spDCM, modeling bidirectional endogenous (**A**‐matrix) connections among all nodes, was fitted to the empirical cross‐spectral density of the BOLD signal using variational Bayesian inversion, yielding posterior distributions of Bayesian parameter averages (BPA) (G. Li et al. 2020). Group‐level inference employed a two‐stage parametric empirical Bayes (PEB) framework. First, a model encoding diagnosis (HC = 0, PSD = 1) tested for disease‐related alterations in effective connectivity, adjusting for age, sex, and years of education. Second, within the PSD group, mean‐centered HDRS scores were included as a covariate to assess symptom‐connectivity associations. Only connections meeting both criteria were reported: (i) strong statistical evidence (*Pp* > 99%, Bayes factor > 100); and (ii) a biologically meaningful effect size (|*β*| > 0.11 Hz), a threshold consistent with prior spDCM studies (Ten Doesschate et al. 2023).

Demographic and clinical characteristics were analyzed using IBM SPSS Statistics version 24.0 (IBM Corp., Armonk, NY, USA). Continuous variables are expressed as mean ± standard deviation (SD), and categorical variables as frequencies. Normality was assessed using the Shapiro–Wilk test. Between‐group comparisons for continuous variables were performed using independent two‐sample *t*‐tests when normality and homogeneity of variance assumptions were met; otherwise, the Mann–Whitney *U* test was applied. Categorical variables were compared using the chi‐square (*χ* ^2^) test. Statistical significance was defined as a two‐tailed *p*‐value < 0.05.

## 3 Results

### 3.1 Participants

The final sample comprised 16 patients with PSD and 14 age‐, sex‐ and years of education‐matched HC. Groups did not differ significantly in sex distribution (PSD: 8 male/8 female; HC: 4 male/10 female; *p* = 0.284), age (PSD: 63.56 ± 9.68 years; HC: 55.43 ± 12.33 years; *p* = 0.053), or years of education (PSD: 9.56 ± 1.59; HC: 11.07 ± 4.45; *p* = 0.246). All PSD patients exhibited mild to moderate depressive symptoms (HDRS: 9.63 ± 2.19) (Table 1).

**TABLE 1:** Demographic features of PSD and HC.

	PSD	HC	*p*
Sex (male/female)	8/8	4/10	0.284
Age (years)	63.56 ± 9.68	55.43 ± 12.33	0.053
Education years	9.56 ± 1.59	11.07 ± 4.45	0.246
HDRS	9.63 ± 2.19	—	—

Abbreviations: HC, healthy controls; HDRS, Hamilton Depression Rating Scale; PSD, post‐stroke depression.

### 3.2 Genetic Overlap Between Stroke and Depression

**FIGURE 2:**
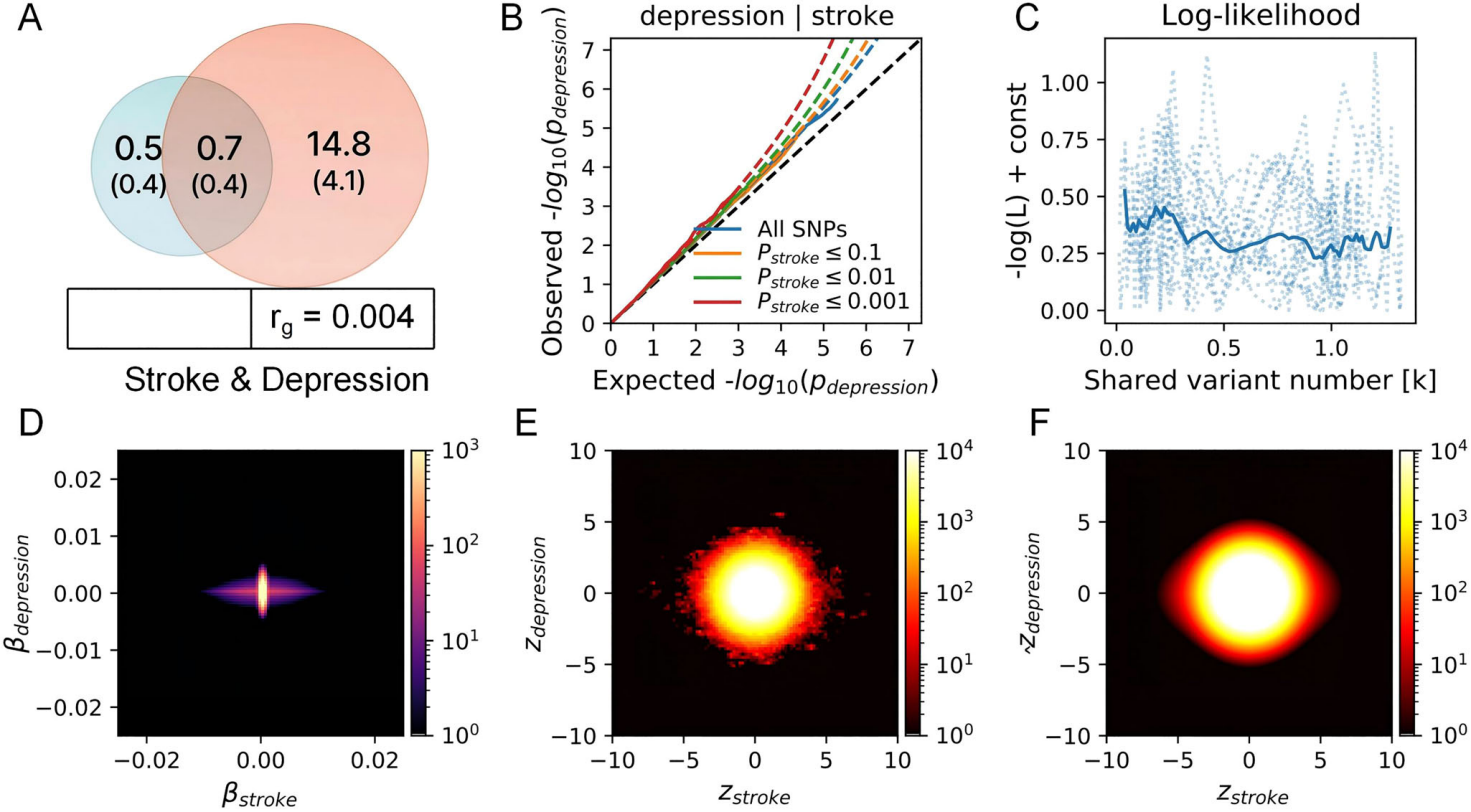
Multiscale dissection of shared genetic architecture between stroke and depression using MiXeR. (A) Venn diagram depicting estimated numbers (in thousands) of non‐null causal variants for stroke (left), depression (right), and their overlap (center). Circle areas are proportional to polygenicity. The genome‐wide genetic correlation (*r*_g_) is shown below, with sign indicating directionality of shared effects. (B) Conditional quantile–quantile (Q–Q) plot of depression GWAS −log_10_(*P*) values stratified by stroke association strength (*p*_stroke_ ≤ 0.1, ≤ 0.01, ≤ 0.001; colored curves) versus all SNPs (gray). Deviation from the null (diagonal) indicates enrichment of depression signals among stroke‐associated variants. (C) Negative log‐likelihood profile (−log L + const) across candidate numbers of shared causal variants (*k*). The blue curve shows the likelihood trend; points represent evaluated *k* values. (D) Joint density of additive causal effect sizes (*β*) for stroke (*x*‐axis) and depression (*y*‐axis) inferred from the fitted MiXeR model. Color intensity reflects the density of SNP effect combinations. (E) Observed joint density of GWAS *Z*‐scores for stroke (*Z*_stroke_, *x*‐axis) and depression (*Z*_depression_, *y*‐axis). (F) Model‐predicted joint density of standardized *Z*‐scores for stroke (*x*‐axis) and depression (*y*‐axis) from the best‐fit MiXeR model. Concordance between (E) and (F) validates model fit.

Univariate MiXeR analysis revealed distinct polygenic architectures: stroke showed low SNP‐based heritability (*h* ^2^SNP = 0.012) and modest polygenicity (1127 variants), whereas depression exhibited slightly higher heritability (*h* ^2^SNP = 0.015) but substantially greater polygenicity (15,496 variants). Despite this disparity, multivariate modeling uncovered a measurable, albeit modest, genetic overlap—quantified by a Dice coefficient of 9% and a weak positive genetic correlation (*r*_g_ = 0.004). Model‐based inference estimated approximately 656 shared variants between the disorders. Strikingly, only 53.4% exhibited concordant directions of effect, revealing a complex landscape of antagonistic and synergistic pleiotropy—wherein many shared loci exert opposing influences on stroke and depression risk (Figure 2).

To identify specific pleiotropic loci, we applied condFDR analysis, leveraging stroke genetics to boost discovery power for depression. This approach identified 18 genomic loci significantly associated with depression conditional on stroke status (condFDR < 0.01), with enrichment on Chromosomes 1 and 4 (Table S1). Integration via FUMA prioritized five high‐confidence shared risk SNPs (Table S2)—providing molecular anchors for cross‐disorder pathophysiology and candidate targets for functional validation.

### 3.3 Tissue‐and Spatially Resolved Convergence of Stroke–Depression Genetic Risk

**FIGURE 3:**
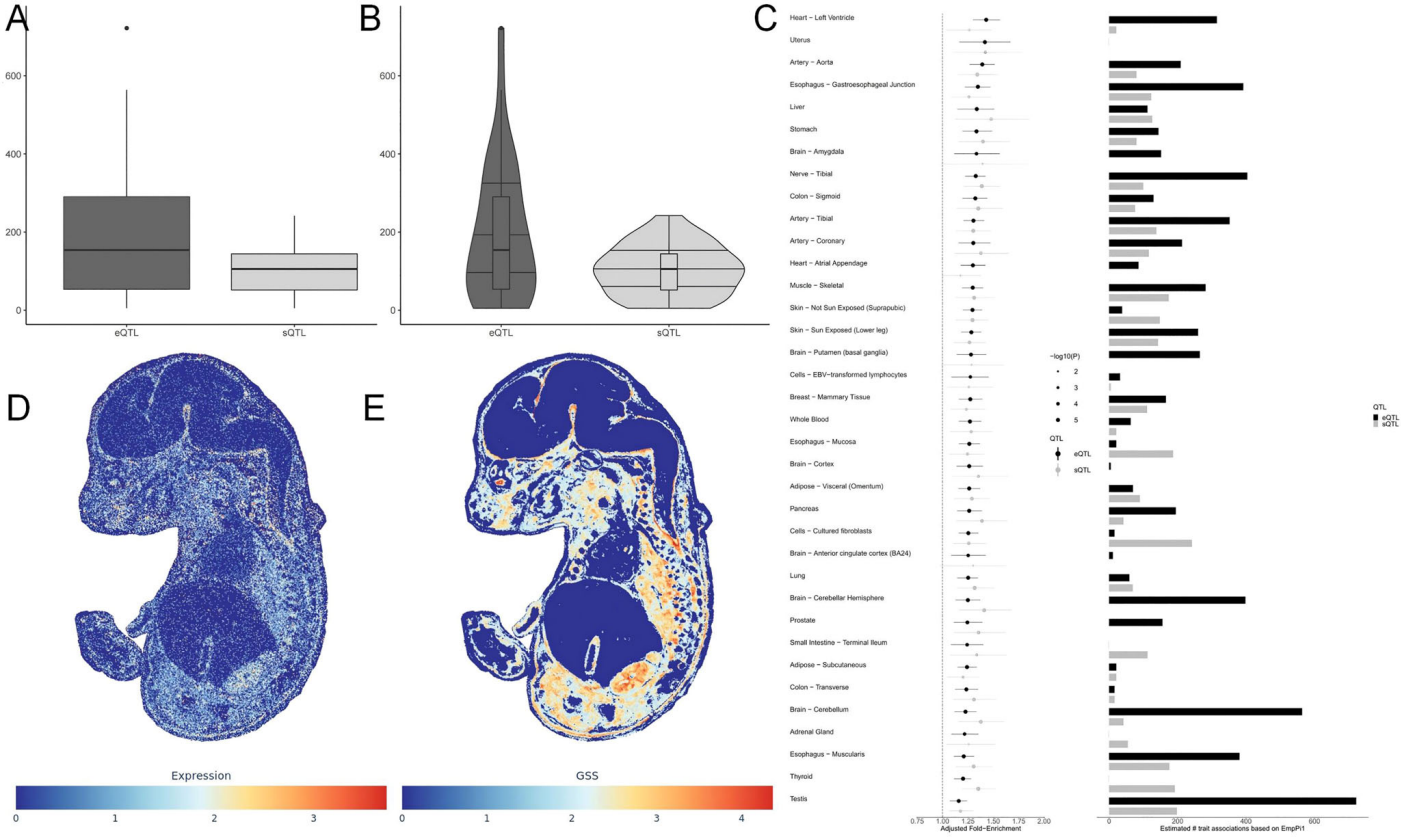
Tissue‐ and spatially resolved enrichment of shared genetic risk for stroke. (A) Boxplots showing enrichment strength (adjusted fold enrichment) of expression quantitative trait loci (eQTLs) and splicing QTLs (sQTLs) across 49 GTEx v8 tissues for stroke GWAS signals. Boxes denote interquartile range (IQR), horizontal lines indicate medians, and points represent outliers. (B) Violin plots integrating kernel density estimates with boxplot elements to illustrate the distribution and dispersion of eQTL and sQTL enrichment across tissues. (C) Paired tissue enrichment profiles comparing eQTL and sQTL significance (−log_10_(*P*)) and adjusted fold enrichment (dot size) across GTEx tissues. Only tissues passing multiple‐testing correction are shown. (D) Spatial mapping of stroke GWAS signals onto the E16.5 mouse embryonic brain using gsMap. Color intensity (blue to red) reflects regional gene expression levels of stroke‐associated genes. (E) Gene set scoring (GSS) map from gsMap, where color intensity indicates the degree of enrichment of stroke GWAS signals in anatomically defined regions of the E16.5 mouse brain.

**FIGURE 4:**
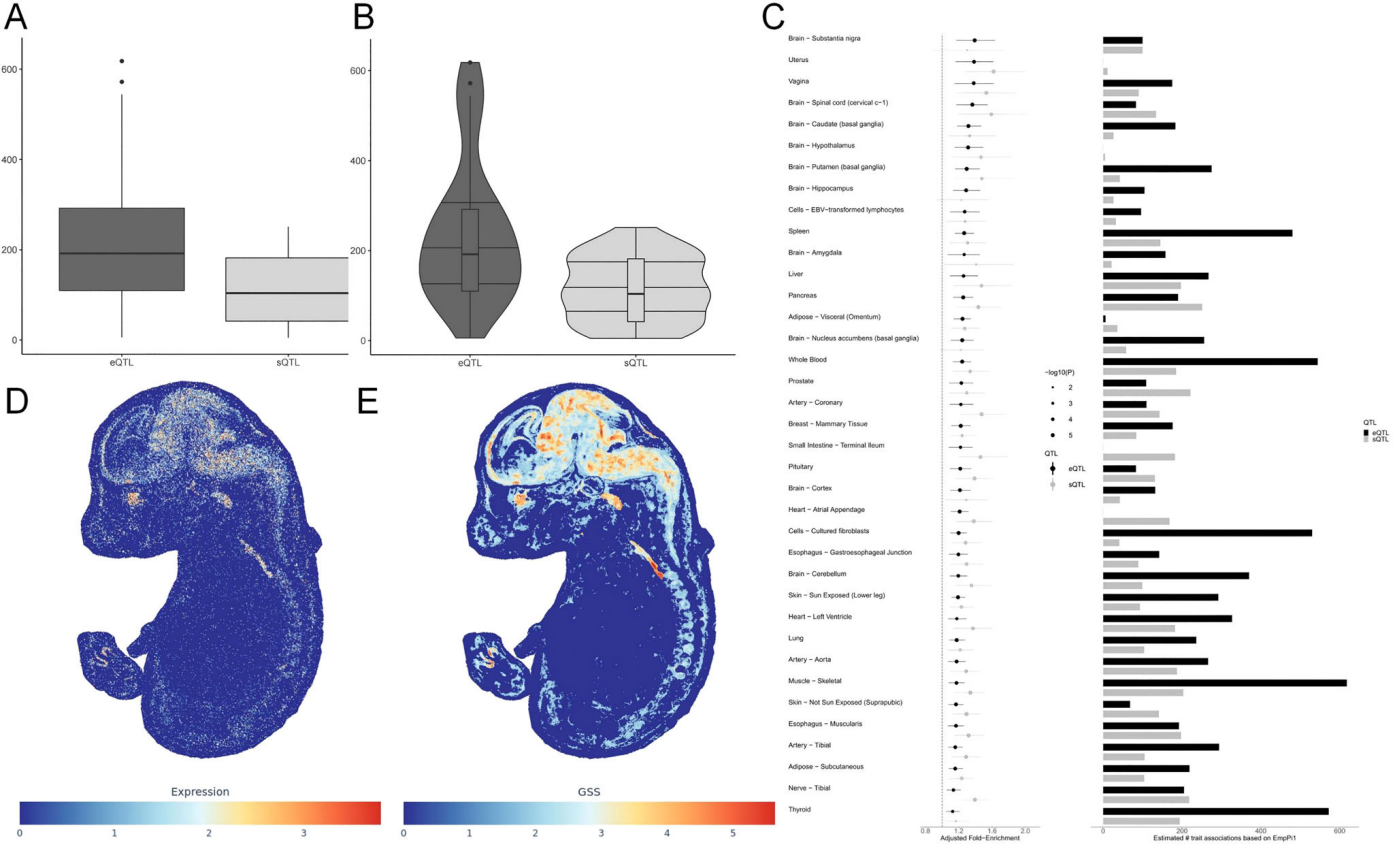
Tissue‐ and spatially resolved enrichment of shared genetic risk for depression. (A) Boxplots showing enrichment strength (adjusted fold enrichment) of expression quantitative trait loci (eQTLs) and splicing QTLs (sQTLs) across 49 GTEx v8 tissues for depression GWAS signals. Boxes denote interquartile range (IQR), horizontal lines indicate medians, and points represent outliers. (B) Violin plots integrating kernel density estimates with boxplot elements to illustrate the distribution and dispersion of eQTL and sQTL enrichment across tissues. (C) Paired tissue enrichment profiles comparing eQTL and sQTL significance (−log_10_(*P*)) and adjusted fold enrichment (dot size) across GTEx tissues. Only tissues passing multiple‐testing correction are shown. (D) Spatial mapping of depression GWAS signals onto the E16.5 mouse embryonic brain using gsMap. Color intensity (blue to red) reflects regional gene expression levels of depression‐associated genes. (E) Gene set scoring (GSS) map from gsMap, where color intensity indicates the degree of enrichment of depression GWAS signals in anatomically defined regions of the E16.5 mouse brain.

To pinpoint convergence of shared genetic risk, we first tested for eQTL/sQTL enrichment across 49 human tissues (GTEx v8) using QTLEnrich. After stringent correction for multiple comparisons and genomic confounders, both stroke and depression showed significant enrichment exclusively in central nervous system tissues—most prominently the dorsolateral prefrontal cortex (DLPFC) and broader cortical regions—implicating dysregulated gene expression and RNA processing in these areas as joint contributors to disease susceptibility (Tables S3 and S4; Figures 3C and 4C). We next applied gsMap—a genetically informed, cross‐species spatial mapping framework—to project GWAS signals onto a high‐resolution transcriptomic atlas of the E16.5 mouse embryonic brain. gsMap simultaneously quantifies (i) regional enrichment of trait‐associated gene expression and (ii) localized convergence of polygenic risk via gene set scoring (GSS). Both analyses revealed striking colocalization of stroke‐and depression‐related genetic risk within discrete neuroanatomical domains, particularly nascent cortical structures during early neurodevelopment (Figures 3D,E and 4D,E).

### 3.4 Disrupted Sensorimotor–Default Mode Coupling in Post‐stroke Depression

**FIGURE 5:**
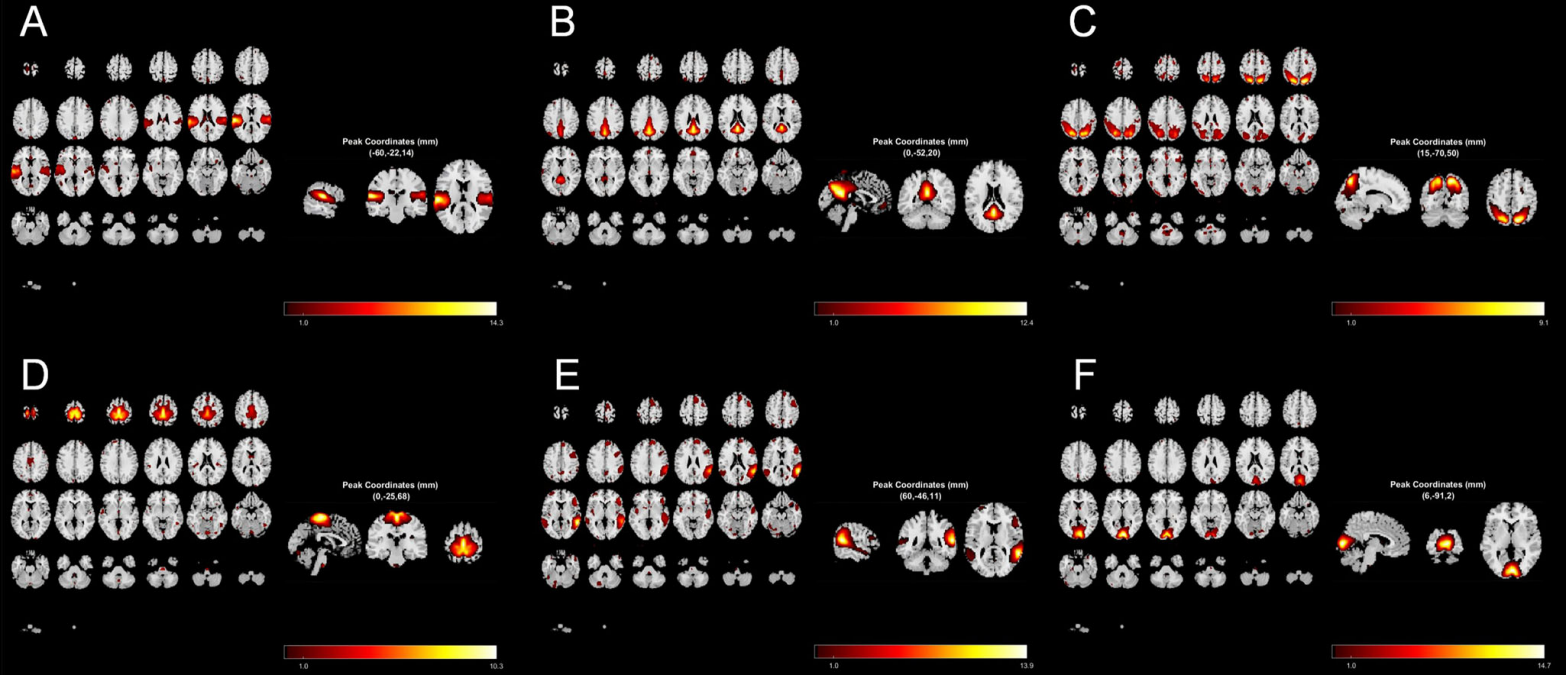
Spatial maps of six neuroanatomically plausible intrinsic connectivity networks identified by group ICA. Networks include: (A) A (AN; IC30, *β* = 0.59), (B) default mode (DMN; IC21, *β* = 0.41), (C) dorsal attention (DAN; IC64, *β* = 0.32), (D) sensorimotor (SMN; IC55, *β* = 0.38), (E) ventral attention (VAN; IC51, *β* = 0.34), and (F) visual (VN; IC43, *β* = 0.44). Each map shows the group‐level spatial *z*‐scored activation pattern (color intensity reflects signal strength). Peak MNI coordinates and corresponding *z*‐statistics are indicated for major hubs.

Group ICA of resting‐state fMRI data identified 64 spatially ICs. After stringent exclusion of artifacts (e.g., motion, vascular noise) and alignment to canonical RSN templates via spatial regression (*β* > 0.3), six neuroanatomically plausible networks were retained: AN (IC30, *β* = 0.59), DMN (IC21, *β* = 0.41), DAN (IC64, *β* = 0.32), SMN (IC55, *β* = 0.38), VAN (IC51, *β* = 0.34), and VN (IC43, *β* = 0.44) (Figure 5).

**FIGURE 6:**
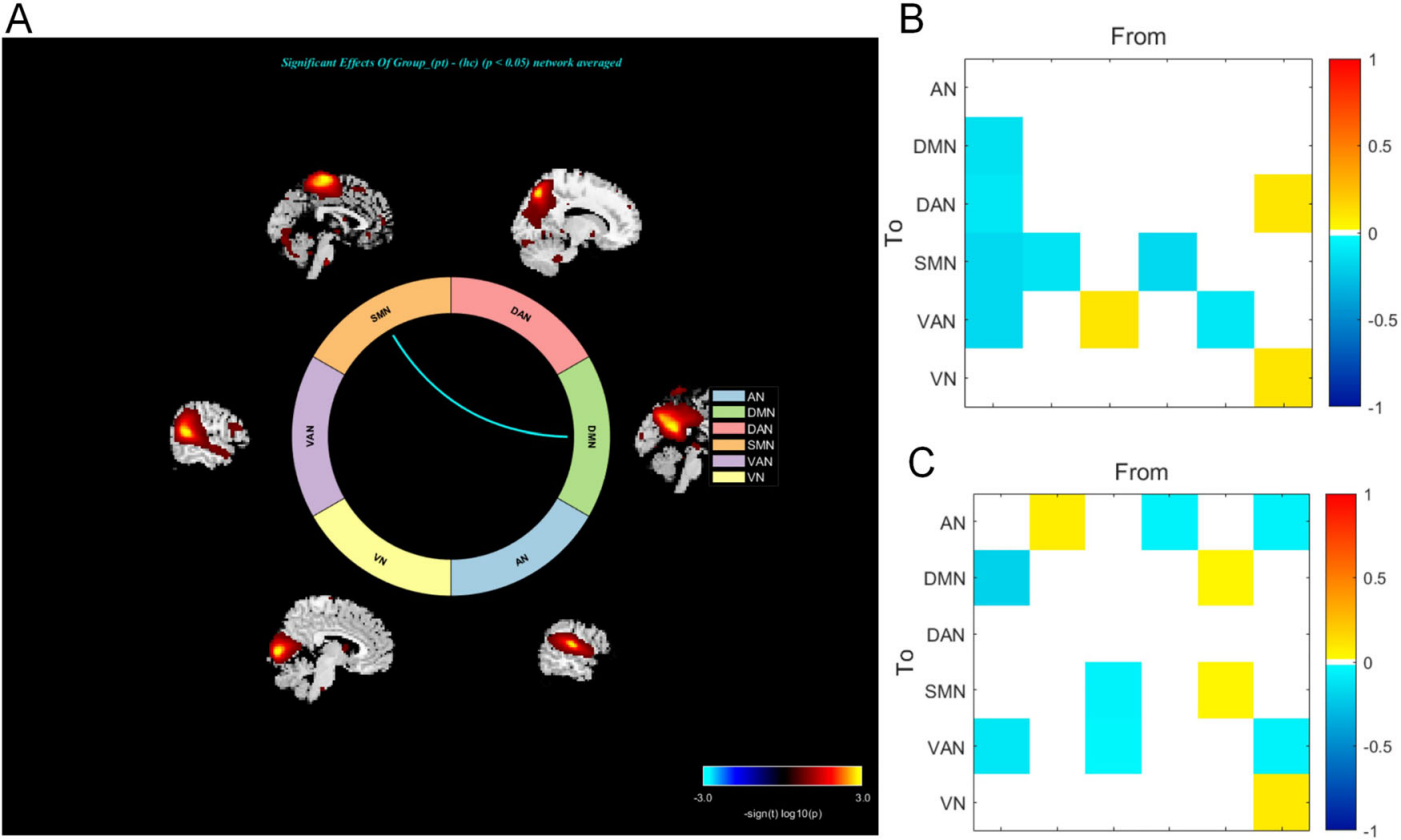
Convergent evidence from undirected and directed connectivity analyses reveals circuit‐level pathophysiology in post‐stroke depression (PSD). (A) Functional network connectivity (FNC) showing significantly reduced coupling between the default mode (DMN) and sensorimotor (SMN) networks in PSD versus healthy controls (FDR‐corrected *q* < 0.05). (B) Spectral dynamic causal modeling (spDCM) reveals weakened top‐down effective connectivity from DMN to SMN (BPA = –0.12 Hz) and broadly attenuated outflow from the auditory network (AN) to DMN (–0.14 Hz), SMN (–0.16 Hz), and ventral attention network (VAN, –0.16 Hz)—all with posterior probability > 0.99. (C) Within the PSD group, greater depression severity (higher HDRS scores) is associated with increasingly negative AN→DMN effective connectivity (BPA = –0.19 Hz, posterior probability > 0.99), establishing this directed pathway as a quantitative biomarker of symptom burden.

Pairwise functional connectivity between these networks revealed widespread dysregulation in PSD relative to HC. Critically, only one connection survived rigorous correction for multiple comparisons (FDR, *q* < 0.05): hypoconnectivity between the SMN and DMN (Figure 6A). This finding highlights a selective decoupling of motor and introspective systems as a core feature of PSD pathophysiology.

### 3.5 Directed Circuit Disruption Underlies Post‐stroke Depression

Bayesian random‐effects model comparison revealed decisive evidence (*Pp* > 0.99) for disrupted effective connectivity in PSD versus HC. PSD was characterized by weakened DMN→SMN influence (BPA = –0.12 Hz, *Pp* > 0.99), reflecting impaired coupling between introspective and motor systems. The AN showed consistently reduced outflow to the DMN (–0.14 Hz), SMN (–0.16 Hz), and VAN (–0.16 Hz; all *Pp* > 0.99), indicating a broad deficit in auditory‐mediated network coordination (Figure 6B). Notably, within PSD, greater depression severity (HDRS) correlated with stronger inhibitory AN→DMN connectivity (BPA = –0.19 Hz, *Pp* > 0.99; Figure 6C), linking symptom intensity to circuit‐level dysfunction. Model fidelity was high (spDCM explained 74%–97% of BOLD variance), confirming robust neural dynamic capture.

## 4 Discussion

Our findings delineate a multiscale architecture underlying the comorbidity of stroke and depression, encompassing shared genetic risk, developmentally anchored cortical vulnerability, and acquired circuit dysfunction. By integrating cross‐disorder genomics, spatial transcriptomic mapping, and causal network modeling, we demonstrate that genetic overlap between stroke and depression is spatially organized, with shared risk variants converging in developing cortical circuits and later manifesting as directed dysconnectivity among the AN, DMN, and SMN. Notably, effective connectivity from the AN to the DMN scaled with depressive symptom severity, identifying this pathway as a candidate symptom‐linked potential biomarker. Together, these results provide a mechanistic framework for PSD and offer a foundation for circuit‐informed potential biomarkers and targeted neuromodulation.

Integrative genomic analyses reveal substantial yet directionally heterogeneous polygenic overlap between stroke and depression. MiXeR estimates that approximately 9% of common variants are shared, but only 53.4% exhibit concordant effects—evidence of widespread antagonistic pleiotropy that masks genetic correlation in LD‐score regression (*r*_g_ = 0.004). By quantifying total variant sharing irrespective of direction, MiXeR uncovers hidden heritable pleiotropy (Shadrin et al. 2025), suggesting clinical comorbidity arises from partially shared genetic liability rather than purely sequential pathogenesis. At condFDR < 0.01, we identified five genome‐wide significant pleiotropic SNPs. Notably, rs2107595 in HDAC9 is a canonical stroke locus (Malhotra et al. 2019). HDAC9 dysregulation contributes peripherally to vascular inflammation and atherosclerosis, increasing ischemic stroke risk (Bellenguez et al. 2012)—emerges as a cross‐disorder hub: peripherally driving vascular inflammation and atherosclerosis, and centrally promoting neuroinflammation, synaptic dysfunction, and neuronal vulnerability linked to depression (Das and Natarajan 2020). Given the high metabolic demand and vascular sensitivity of thalamocortical auditory pathways, we hypothesize that HDAC9‐mediated neuroinflammation may preferentially compromise the structural integrity of these connections, thereby disrupting AN function (Lisek et al. 2025). In addition, rs1906618 near PITX2, a master regulator of cardiac development, shows robust cross‐trait association (Chen et al. 2025; Ikenouchi et al. 2024) and is newly implicated in neurodevelopmental patterning and circuit maturation (Khamkar et al. 2025). Similarly, PITX2‐driven alterations in circuit maturation could disrupt the long‐range wiring required for AN–DMN integration. Together, these loci implicate convergent inflammatory and cardio‐cerebral developmental pathways as shared molecular substrates of stroke–depression comorbidity (Bordon 2025). Critically, this shared genetic burden may constrain poststroke neural plasticity (Lindgren 2025). Aligning with current literature on maladaptive plasticity in mood disorders, we propose that neurodevelopmental risks limit the brain's capacity to reorganize vulnerable circuits following injury, thereby stabilizing the AN→DMN disruption as a persistent pathological trait rather than a transient state.

A defining feature of the shared genetic architecture between stroke and depression is its non‐uniform spatial patterning across the brain. While depression showed widespread heritability enrichment across association cortices—as expected for a disorder involving distributed mood‐regulatory circuits—stroke exhibited focal enrichment specifically in the DLPFC, a hub for executive control and cognitive‐emotional integration (Kang et al. 2012). Critically, the DLPFC represents a selective neuroanatomical interface where cerebrovascular vulnerability and mood‐related genetic risk converge. This colocalization does not imply that both disorders are equally DLPFC‐driven; rather, it suggests that shared variants may dysregulate gene expression (e.g., via eQTLs/sQTLs) in this region, simultaneously increasing susceptibility to vascular injury and impairing top‐down emotional regulation (de Klein et al. 2023). Thus, the DLPFC emerges as a spatially grounded nexus explaining their clinical comorbidity (Pan et al. 2022). Clinically, this DLPFC‐centered vulnerability aligns with the core phenotype of PSD—characterized by persistent low mood, anhedonia, psychomotor retardation, impaired concentration, and fatigue—symptoms reflecting disrupted cognitive‐emotional integration. In severe cases, agitation, anxiety, or suicidal ideation may accompany this syndrome (W. Lu and Wen 2024).

Our findings support a developmental origin for the shared etiology of stroke and depression. Projecting GWAS signals onto a spatially resolved E16.5 mouse embryonic brain transcriptome (gsMap) revealed significant enrichment of shared genetic risk in nascent cortical regions, including precursors of the DLPFC (Song et al. 2025)—extending disease vulnerability to critical windows of embryonic neurodevelopment (Major Depressive Disorder Working Group of the Psychiatric Genomics Consortium 2025). We propose that common risk variants perturb neural progenitor dynamics, establishing latent cortical vulnerabilities that remain silent until unmasked by a second hit (Schork et al. 2019). In adulthood, a severe cerebrovascular insult—such as ischemic stroke—triggers decompensation of this primed circuitry (Dixon and Muotri 2023), unleashing a cascade that amplifies depressive pathophysiology and culminates in network dysfunction and clinical symptoms. This spatiotemporal “two‐hit” model positions embryonic neurodevelopment as the substrate of genetic risk and stroke as the precipitant of phenotypic expression.

In PSD versus HC, only one inter‐network coupling survived stringent multiple‐comparison correction: DMN–SMN hypoconnectivity. spDCM confirmed a specific deficit in directed influence from DMN to SMN (BPA = –0.12 Hz, *Pp* > 0.99), pinpointing impaired transmission of internally oriented cognition to sensorimotor systems—a core pathophysiological feature of PSD that may underlie psychomotor retardation (Dahms et al. 2024; Kaiser et al. 2015). Beyond this dyad, the AN exhibited broadly attenuated effective outflow to the DMN, SMN, and VAN—all *Pp* > 0.99—revealing a systemic failure in auditory‐mediated coordination across introspective, motor, and attentional domains (Jellinger 2021). Critically, AN→DMN connectivity scaled linearly with symptom severity: more negative coupling (BPA = –0.19 Hz, *Pp* > 0.99) robustly predicted higher HDRS scores. Mechanistically, this association may reflect impaired processing of emotional prosody (Thomasson et al. 2021), where the AN fails to relay affective auditory cues to the DMN for self‐referential integration. Furthermore, given the AN's structural connections with limbic regions (e.g., amygdala), this disruption could bias internal cognition toward negative valence, a core feature of depression exacerbated by stroke‐related sensory deficits (Wen et al. 2023). While auditory–limbic dysregulation is implicated in depression (Wang et al. 2024; J. Zhang et al. 2023), our findings establish AN→DMN as a PSD‐specific, symptom‐dimensioned potential biomarker. These results position aberrant auditory–DMN communication not as an epiphenomenon, but as a mechanistically grounded target for circuit‐informed neuromodulation in PSD (Korgaonkar et al. 2020).

Several limitations merit acknowledgment. First, due to the cross‐sectional design, we cannot infer causal relationships between genetic risk, circuit disruption, and depression severity. Second, genetic analyses faced four constraints: (i) limited depression GWAS power may have yielded a conservative overlap estimate (Dice = 0.09), indicating shared risk co‐occurs with disorder‐specific factors; (ii) European‐ancestry predominance limits generalizability and masks ancestry‐specific architectures; (iii) cross‐species mapping relies on developmental homology assumptions, where species‐specific trajectories may affect spatial localization accuracy; and (iv) the current framework does not account for environmental exposures or gene–environment interplay, which are established modulators of depression‐related brain phenotypes (F. Liu et al. 2023; Z. Zhang et al. 2025). Third, functional annotation relied on eQTL/sQTL maps from neurotypical postmortem brains, which cannot capture disease‐state‐dependent regulatory dynamics in stroke, depression, or their interaction. Fourth, restriction to mild‐to‐moderate depression limits generalizability to severe PSD. Consequently, without independent replication, the AN→DMN connectivity pattern represents a potential rather than definitive biomarker. Future multicenter, longitudinal studies with broader severity ranges are warranted to validate its clinical utility. Despite these constraints, convergent evidence across genomic, spatially resolved transcriptomic, and directed circuit levels establishes a robust, multiscale framework for mechanistic investigation.

## 5 Conclusion

Our multiscale analysis redefines PSD as a syndrome arising from shared developmental genetic risk and acquired circuit failure. Common variants—enriched in nascent cortical regions during embryogenesis—establish latent vulnerability in prefrontal circuits, which is unmasked by a later‐life stroke. This “two‐hit” cascade culminates in directed decoupling of introspective (DMN) and sensorimotor (SMN) systems, compounded by AN dysregulation that scales precisely with symptom severity. Critically, AN→DMN effective connectivity emerges as a PSD‐specific, symptom‐dimensioned potential biomarker. Together, these findings nominate high‐confidence targets for mechanism‐based intervention: HDAC9‐mediated neurovascular inflammation, PITX2‐driven cardio‐cerebral crosstalk, and AN–DMN circuitry—providing a roadmap for diagnostics and circuit‐informed therapeutics.

## Author Contributions

**Zhi‐Jie Xu**: conceptualization, formal analysis, investigation, methodology, writing – original draft. **Su‐Xiang Zhang**: data curation, formal analysis, investigation, methodology, writing – original draft. **Ji‐Ling Li**: formal analysis, investigation, methodology, software, writing – original draft. **Jia‐Jia Wu**: formal analysis, funding acquisition, validation. **Jie Ma**: formal analysis, funding acquisition, validation. **Zhen‐Zhen Ma**: data curation and investigation. **Ji‐Ming Tao**: project administration, resources, supervision, writing – review and editing. **Xu‐Yun Hua**: funding acquisition, investigation, project administration, resources, supervision, writing – review and editing. **Jian‐Guang Xu**: conceptualization, funding acquisition, project administration, resources, supervision, writing – review and editing.

## Funding

This work was supported by the National Natural Science Foundation of China (Grant Nos.: 82272583, 82172554, 82272589, and 82302870; the Shanghai Health Care Commission (Grant No.: 2022JC026); the High‐Level Chinese Medicine Key Discipline Construction Project (Integrative Chinese and Western Medicine Clinic) of the National Administration of TCM (Grant No.: zyyzdxk‐2023065); and the Shanghai Hospital Development Center Foundation‐Shanghai Municipal Hospital Rehabilitation Medicine Specialty Alliance (Grant No.: SHDC22023304).

## Ethics Statement

The study was approved by the Shuguang Hospital Ethics Committee (No. 20241419‐002‐01).

## Conflicts of Interest

The authors declare no conflicts of interest.

## Supporting information




**Supplementary Table**: brb371502‐sup‐0001‐TableS1‐S4.xlsx

## Data Availability

Publicly available GWAS summary statistics were obtained from the consortia repositories detailed in Section 2. The following researcher‐generated materials are available from the corresponding author upon reasonable request: (i) derived neuroimaging measures (e.g., subject‐specific regional BOLD time series); (i) statistical outputs (e.g., PEB parameter estimates and effective connectivity matrices); and (iii) analysis scripts for data preprocessing, spDCM modeling, and statistical inference (implemented in MATLAB/SPM12). All materials will be shared in standardized formats within 30 days of request, subject to institutional data‐use agreements and ethical approvals.
